# Semantic-Aware Security Orchestration in SDN/NFV-Enabled IoT Systems

**DOI:** 10.3390/s20133622

**Published:** 2020-06-27

**Authors:** Alejandro Molina Zarca, Miloud Bagaa, Jorge Bernal Bernabe, Tarik Taleb, Antonio F. Skarmeta

**Affiliations:** 1 Department of Information and Communications Engineering, University of Murcia, 30100 Murcia, Spain; alejandro.mzarca@um.es (A.M.Z.); skarmeta@um.es (A.F.S.); 2 Communications and Networking Department, School of Electrical Engineering, Aalto University, 02150 Espoo, Finland; miloud.bagaa@aalto.fi (M.B.); tarik.taleb@aalto.fi (T.T.); 3 Centre for Wireless Communications (CWC), University of Oulu, 90570 Oulu, Finland; 4 Department of Computer and Information Security, Sejong University, Seoul 05006, Korea

**Keywords:** security, semantic technologies, SDN, NFV, QoS, optimization, IoT

## Abstract

IoT systems can be leveraged by Network Function Virtualization (NFV) and Software-Defined Networking (SDN) technologies, thereby strengthening their overall flexibility, security and resilience. In this sense, adaptive and policy-based security frameworks for SDN/NFV-aware IoT systems can provide a remarkable added value for self-protection and self-healing, by orchestrating and enforcing dynamically security policies and associated Virtual Network Functions (VNF) or Virtual network Security Functions (VSF) according to the actual context. However, this security orchestration is subject to multiple possible inconsistencies between the policies to enforce, the already enforced management policies and the evolving status of the managed IoT system. In this regard, this paper presents a semantic-aware, zero-touch and policy-driven security orchestration framework for autonomic and conflict-less security orchestration in SDN/NFV-aware IoT scenarios while ensuring optimal allocation and Service Function Chaining (SFC) of VSF. The framework relies on Semantic technologies and considers the security policies and the evolving IoT system model to dynamically and formally detect any semantic conflict during the orchestration. In addition, our optimized SFC algorithm maximizes the QoS, security aspects and resources usage during VSF allocation. The orchestration security framework has been implemented and validated showing its feasibility and performance to detect the conflicts and optimally enforce the VSFs.

## 1. Introduction

As the Internet of Things (IoT) is being extensively adopted in our society, the security issues associated are increasing accordingly. The heterogeneity, pervasiveness, and constrained nature of IoT devices and IoT network protocols are paving the way to an increasing number of security threats and attacks [1,2]. In this sense, Network Function Virtualization (NFV) and Software-Defined Networking (SDN) can help to manage, optimize and dynamically mitigate cyberattacks and threats enhancing the overall resilient capabilities of IoT systems. To this aim, NFV can rely on virtualization and Cloud computing techniques to provide elastic capabilities needed to achieve a fast reaction and recovery from cyberattacks. In this sense, lightweight Virtual network Security Functions (VSF) such as vFirewalls [3], vAAA [4], vIDS or vIoT-Honeynet [5] can be dynamically allocated and orchestrated in the IoT domain, while the SDN approach can help to dynamically (re)configure the network by software, splitting the control and data planes.

This security management can be driven by orchestration policies and security intents, that can be translated into security configurations to configure, manage and deploy the associated VSFs and VNFs across edges, fog nodes and Cloud data centers. The benefits of this approach are manifold, as it enables not only on-demand security provisioning, but also dynamic countering of cyberattacks depending on the actual context, thereby providing self-protection and resilient capabilities to the IoT managed system.

Nonetheless, Orchestration and Service Function Chaining (SFC) of those virtual security functions can derive new attack vectors, misconfigurations and conflicts with regard to the managed system and the management policies ruling the VSFs. Despite the latest advances to manage automatic security management in SDN/NFV-aware systems [6,7], those solutions do not formally deal with the potential semantic conflicts that might occur between the security orchestration of diverse security policies and actual system context. Besides, actual SFC approaches do not consider security aspects to compute the optimal chaining, as they tend to focus only on the optimization of end-to-end quality of service (QoS), and compute resources in the service chaining [8,9].

To fill this gap, this paper presents a semantic-aware policy-driven orchestration mechanism for autonomic and verifiable security management and VSF Service Function Chaining (SFC) in softwarized and virtualized IoT scenarios. In our proposal, the IoT system is continuously monitored generating an instantiated data model that is maintained in real-time in a Knowledge Base (KB). The system model has been modeled using the logic formalism provided by the Semantic Web technology, which enables the usage of reasoners to infer new knowledge and formal verification of the entire orchestration operation. Our semantic reasoner is able to perform a formal validation and verification of the IoT model, whereas the rule-reasoning fed by horn-like rules allows to derive additional information not explicitly defined in the model. Thus, the usage of Semantic technologies and rule reasoning, allows different ways to detect conflicts that may appear in the IoT system model and policies. This provides a remarkable added value for the security policy orchestration not found yet in the research literature, such as the detection of semantic conflicts that might occur among the instantiated system model and the orchestration security policies to be chained.

In addition, the proposed orchestration framework is able to optimize the deployment and life-cycle management of VSF according to the policy specifications, through and algorithm that maximizes the security guarantees in network chaining and QoS, while minimizing the cost’s deployments. The Security Orchestrator relies on the policy interpreter component in order to check the validity and consistency of the orchestration policies prior to allocating the associated VSF. Besides, the Orchestrator performs a context-based rule reasoning, i.e., a formal validation of the syntactic and semantic facts in the KB, looking for conflicts between the already enforced security policies, the newly added ones and the contextual IoT network and system situation. This process allows verifying that the new policies are enforceable considering the current system and networks status, the expected versus available security levels, time and other system conditions, as well as available security enablers, i.e., available VSF.

Our semantic-aware based security orchestration mechanism has been implemented and validated in a real testbed, showing his feasibility and performance to efficiently verify the consistency of the orchestration policies and generate optimal SFC chaining of virtual security appliances by considering QoS, actual resources and security conditions.

The rest of this paper is structured as follows. Section 2 presents the related work. Section 3 is devoted to the general security management that embraces our security orchestration. Section 4 delves into SFC based efficient security orchestration. Section 5 describes the semantic-aware and policy-based orchestration in IoT systems. Validation, performance evaluation of the implementation and results are presented in Section 6. Finally, Section 7 concludes the paper.

## 2. Related Work

This section describes the state of the art in the three research areas related to this paper, including NFV/SDN-based Security orchestration, SFC chaining optimization and Semantic-based network and security management.

### 2.1. NFV/SDN-Based Security Orchestration in IoT

Security and network Orchestration [10,11] leveraging NFV/SDN in next-generation networks and IoT [12,13], is gaining more and more research attraction as key element to achieve truly efficient, reliable and resilient infrastructures. In this sense, novel zero-touch network security management architectures and frameworks [6,14] for IoT are emerging, which aims to optimize the deployment and (re-)configuration of virtualized and softwarized network security appliances, such as vFirewalls, vHoneynets, vAAA, vChannelProtection, vProxies or vIDS (Intrusion Detection Systems).

In this sense, Zarca et al. [4] manage dynamically virtual AAA as well as security associations (Datagram Transport Layer tunnels) in SDN-Based IoT Networks. Similarly in [3] authors proposed an automatic deployment and configuration of network filtering rules in centralized way by relying on SDN/NFV technologies. In [15], Galeano et al. propose a entropy-based solution to detect and mitigate DDoS Attacks in IoT-Based systems relying on SDN. Likewise, authors in [5] relies on a similar approach for automatic management virtual IoT honeynets to mitigate cyberattacks. However, those research works did not followed a semantic-based approach for the security orchestration, whereby allowing formal verification and conflict detection of the security policies enforced in the system, inferring meaningful conclusions that can be used to drive the orchestration.

### 2.2. VNF or Chestration and SFC Chaining Optimization

In [16] authors analyze the problem of VNFs allocation optimization with optimal algorithms, but they do not actually deal with service chaining and order of VNFs. Likewise, in [17] authors address the service chaining optimization challenge but they do not consider global delays of network traffic in the SFC path. Similarly, in [8] authors formalize the VNF placement and SFC problem and follow a Integer Linear Programming (ILP) to deal with the optimization problem, considering the distance across clients and VNFs and their cost. However, they do not consider other resources and security aspects as addressed in our model. In contrast to this solution, our model considers cost-efficient life cycle management when allocating computational and network resources. In [18], authors address the problem of traffic engineering with multiple paths for an NFV enabled IoT system, configuring dynamically flow tables in virtual switches for managing IoT traffic, considering account variations of traffic volume across time periods. In [9] authors analyze the VNF scheduling and resource allocation with service chain, and provide resource optimization solutions. They define the scheduling decisions and chaining based on service transmission and VNF’s processing delays. However, those previous works, unlike our work they do not consider security aspects in the formulation. A reliability-aware SFC provisioning of VNFs with delay guarantees is proposed in [19]. They suggest a mixed integer linear program (MILP) for VNFs allocation that maximizes reliability and end-to-end delays. Artificial Intelligence is starting to be exploited for VNF scheduling [20]. However, in their work authors only account on transmission and processing delays to optimize the SFC.

### 2.3. Semantic-Based Network and Security Management

Bernabe et al. [21] defined and implemented an authorization system that relies on the logic formalism provided by the Semantic-Web technologies to protect the access to resources in the Cloud. The proposal modelled the underlying infrastructure, the authorization and authentication model, as well as the semantic-web rules to infer authorization decision and detect conflicts. In [22], Sicari et al. proposed a IoT middleware aimed to perform a flexible Security Policy Enforcement of networked smart objects. The proposed system was intended to handled access control (using attribute based encryption), protection of data transmissions through encryption and even with policy violation attempts. In [23], Basile et al. proposed a mechanism for automatic Enforcement of Security Policies in NFV Networks. The proposal defines a refinement mechanism for transformation of high-level security requirements into configuration settings for the network security functions (NSFs). Nonetheless, unlike our work, that paper does not consider inference reasoning to perform conflict detection in the orchestration policies.

Valenza et al. [24] proposed a formal approach for network security policy validation. Like in this paper, they checked the actual network status based on monitoring and configuration, and performed conflict detection analysis to find out misconfigurations or software attacks. Besides, they use remote attestation to validate the trustworthiness of the controls. Nonetheless, they perform the conflict detection across filtering policies, whereas in our paper we present conflict detection mechanisms across different kind of security and orchestration policies. In [25], authors proposed a Inter-function anomaly analysis for correct SDN/NFV deployment. The framework is able to deal with different kinds of network functions, including firewalls, NAT/NAPT devices, traffic monitors and encryption devices, and the model is able to detect anomalies blocked traffic, modified traffic and encrypted traffic. Nonetheless, that paper did not consider the semantic technologies to perform conflict detection in orchestration policies, as it is done in this paper.

## 3. Orchestration Framework Overview

**Figure 1:**
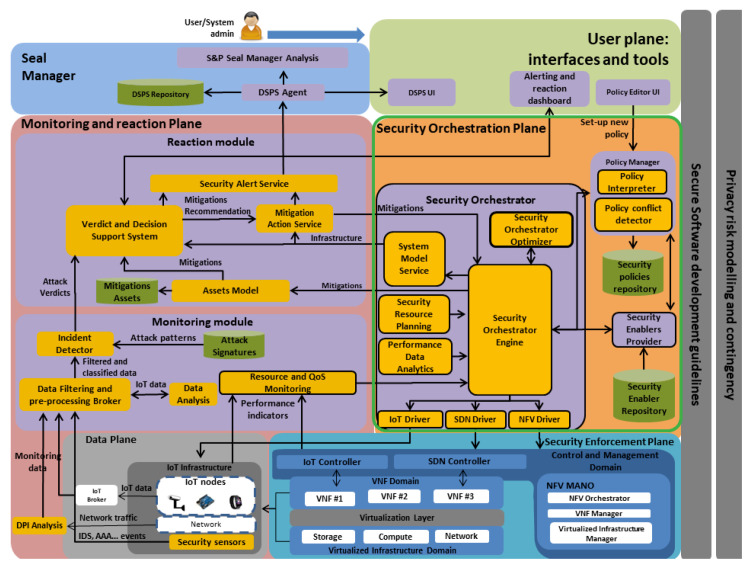
Proposed security Orchestration plane scoped in H2020 ANASTACIA framework.

This section presents the general framework that has been developed within the scope of H2020 ANASTACIA EU project that targets the security management of heterogeneous and distributed IoT applications including smart cities, smart factories and smart buildings. ANASTACIA framework has been developed in a plane-based architecture defined in our previous work [6]. As depicted in Figure 1, the framework consists of distinct planes including: (i) Monitoring and reaction (MR) plane; (ii) Security enforcement (SE) plane; (iii) User plane; (iv) Seal manager (SM) plane; (v) Security orchestration (SO) plane.

This paper focuses on the Security Orchestration plane, and extends our original general IoT management architecture described in [6], by describing the internals of the Security Orchestration plane that were not addressed in that paper, including the orchestration sub-modules, orchestration algorithm’s, process flows and semantic processes required during the orchestration.

The MR plane is responsible for the misbehavior detection that could happen in the network through different monitoring agents. It explores both filtering activities and data analysis for detecting different anomalies. SE plane leverages the strength of SDN technology to interconnect the Physical Network Functions (PNFs), Virtual Network Functions (VNFs) and IoT devices for enabling different IoT services. SE plane will interconnect the cloud domain with IoT domain in the data plane, whereby different IoT services are running. Formally, the communication between a user and an IoT domain happens through a list of chains of VNFs and PNFs named Service Function Chaining (SFCs) which consists of three parts: The ingress point; which is the first VNF in the SFC. The user initially attaches to the ingress point; The intermediate VNFs; The egress point: which is the last VNF in the SFC. The egress point should be connected to the IoT controller.

The order of the communications between the VNFs is defined according to the different SDN rules enforced thanks to the SDN controller. The nature and the size of the SFCs would be defined according to the nature of the user (a normal or a suspicious).

The SE plane is responsible for mitigating the threats either at the PNFs, VNFs or IoT devices by directly interacting at these components and/or the network interconnecting them. The communication between different peers (End-user and IoT devices) happens thanks to VNFs and PNFs installed in the network. When the anomalies are detected by the MR plane, an alert should be generated and forwarded to the SO plane, which mitigates the attacks by dint of SE plane by:

- Pushing different rules at the network by leveraging software defined networking (SDN);
- Interacting at the IoT devices and PNFs by pushing different rules that mitigate the attacks, such as shutting off the malicious IoT devices;
- Creating different VNFs (e.g., virtual firewall) and enforce the traffic to pass through these VNFs, such that the exploit of different vulnerabilities are mitigated through ports communication restriction.

While the user plane offers a policy editor for the administrators to model different security policies with a high/medium level abstraction, the seal manager plane is the responsible for graphically presenting the status of the security and the privacy for the end user.

The SO plane that oversees orchestrating the security enablers according to the security policies generated and forwarded from other framework’s components, such as the MR plane. The security orchestrator relies on a Semantic-aware policy conflict detector to formally validate the consistency of the security policies and orchestration policies. The semantic technologies allow us to represent the semantics of the managed IoT system model and the management policies through rules and facts. Thus, the orchestrator is endowed with a knowledge-based inference engine (expert system) that acts as a reasoner that can infer new knowledge from the models, thereby capturing meaningful information and conclusions. The orchestrator checks any semantic conflict that may appear between security orchestration policies, the already enforced policies held in the Knowledge Base (KB), as well as inconsistencies between the instantiated system model and the new enforced policies.

Moreover, in order to mitigate the various attacks without affecting the QoS in different verticals, the SO plane takes into account the policies requirements and the available resources in the underlying infrastructure. Specifically, it refers to the available amount of resources in terms of CPU, RAM, and storage in different cloud providers including edge and fog nodes, as well as the communication network resources including the end to end bandwidth, jitters and delay. The quality of the link varies according to the locations of different communicating peers (i.e., intra and inter cloud communication), as well as the use of secure channels with different levels including IPsec, Secure Socket Layer (SSL) and Transport Layer Security (TLS).

As depicted in Figure 1, the security orchestration system consists of the following components:

- Resource and QoS Monitoring (RQM) component: this component is part of both MR and SO planes. It is responsible for monitoring the underlying resources used by different VNFs and clouds, as well as the quality of communication between different communication peers in the network;
- Data Analytics (DA) component: this component analyses in real time the monitored SLAs, QoS and available/consumed physical/virtual resources.
- Security Orchestrator Engine (SOE) component: is the core component of the orchestration responsible to mediate between the rest of the components of the orchestration plane.
- Security Orchestrator Optimizer (SOO) component: this component runs the optimization algorithm that infers the best VNF allocation and Service Function Chaining, considering resources and network conditions.
- System model Service: this service is intended to provide an interface to push data and query information from the system model, that is kept up-to-date instantiated in a data base.
- Policy Interpreter (PI): the policy interpreter translates security policies specified in MSPL language to specific security enabler configurations. The policy Interpreter also holds the policy conflict detector aimed to check the consistency and semantic conflicts between the policies and the system model.
- Security Enablers providers (PI): this component provides an interface to access the security enablers that are available in the framework.
- Orchestration Drivers: the security orchestrator is endowed with different purpose drivers used to interact with specific implementations of IoT controllers, SDN Controller and NFV systems. Those drivers allows to automatically enforce the security enablers and their configurations in the managed system.

**Figure 2:**
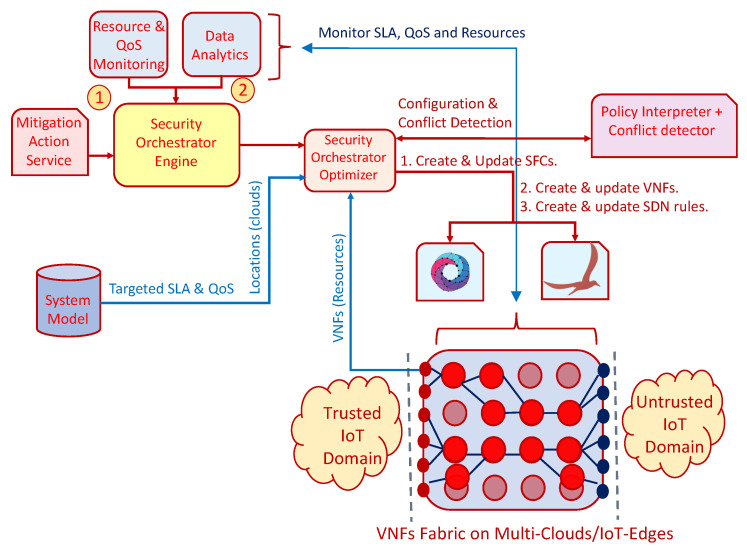
Orchestration system general flow overview.

Our general orchestration mechanism is shown in Figure 2. The data analytics component and the resource monitoring feeds the orchestration engine, while the Orchestrator interacts with the Policy interpreter to accomplish the policy refinement and conflict detection tasks. The algorithm running in the Optimizer creates and updates the SFC to be enforced in the virtualized system through specific controllers (e.g., ONOS) and VNF orchestrators (e.g., OpenMANO). As depicted in Figure 2, the order of the communication between the VNFs is defined according to the different SDN rules enforced, which in turn, are derived from the security policies. The nature and the size of each SFC are determined according to the nature of the user. For suspicious users, more security VNFs would be inserted to filter the traffic and protect the network. As depicted in this figure, the security orchestrator would be triggered either to: (1) the attack detection; (2) the lack in the QoS or disagreement in SLA.

In the former case, an MSPL message is generated and send the orchestration plan from the mitigation action service (MAS) after the successful detection of different attacks. In this case, the security orchestrator optimizer is involved in making a wise decision. The latter would be enforced by selecting the adequate mitigation plane thanks to the SDN controller and the NFV orchestrator. For making such a decision, the security orchestrator needs to consult the system model for getting information about SLA, QoS, deployed VNFs and VSFs, and their locations. The security orchestrator also negotiates with the conflict detector for preventing the deployment of security rules that could create semantic conflicts. After the selection of the adequate security rules (i.e., SFC) that do not generate conflicts with already enforced policies, the security orchestrator interacts with the policy interpreter to translate the received MSPL to the low configuration (LSPL) needed for different enablers including VNFs and SDN rules. The security orchestrator enforces the received LSPL via the NFV orchestrator and the SDN controller. Then, the security orchestrator updates the system model for further use.

In the latter case, in the detection of a drop in the QoS or disagreement in SLA via the “resource & QoS monitoring” component, the security orchestrator will consult the security orchestrator optimizer for redirecting the traffic via existing SFCs or generating new SFC. In both cases, the policy interpreter and conflict detector would be involved in preventing the conflicts and forming LSPL from MSPL. Finally, the LSPL configurations are enforced thanks to the SDN controller and the NFV orchestrator, and then the system model is updated for further use.

### System Model Definition

The IoT system and network model is used as baseline to make contextual-based orchestration decisions, including policy refinement, conflict detection, VNF/VSF allocation and chaining of the associated. The system model is updated continuously in real-time thanks to dedicated monitoring probes that feed the common model across the elements of the framework. For instance, the system model is updated with network topology information coming from the SDN controller. The system model is instantiated, maintained in the a data base, as well as kept synchronized in a knowledge-base used by a semantic rule reasoner that holds the respective instances or facts of the corresponding network elements of the managed IoT system. These individuals are referred in the Security policy models and semantic-based rules, that detect formal inconsistencies in the orchestration policies, and semantic conflicts in the policies deepening on the evolving instantiated the System model. This process will be described in Section 5.

Regarding the system model design, current model allows to represent device networks both physical and virtual as well as their status and the policy enforcements. Device concept has a flavor which represents the hardware specs as well as a location in terms of GPS and human readable values. It also refers to different network interfaces (e.g., 6LoWPANInterface) which model network attributes. Each interface can be involved in multiple connections as source or destination with several flows. Depending on the nature of the device, it can be extended for providing more specific information. For instance, PhyDevice model represents a physical device while VInstance model can be a virtual device (e.g., virtual IoT device), which in turn, can be executed inside a Device (virtual or physical) by using some kind of virtual engine (software). Thus, a device can execute different SoftwareInstances (e.g., snort) that can be configured by applying a set of Configuration parameters. They can also provide different APIs for managing any kind of interaction. Finally, the PolicyTranslation model keeps the traceability among security policies, generated configuration and references to the location where they are instantiated in the infrastructure.

## 4. SFC Based Efficient Security Orchestration System

### 4.1. Network Model and Problem Formulation

In this section, we present the network model and problem formulation of the security orchestrator optimizer used in our security orchestration plane. We denote by G(V∪U,E,W) the network infrastructure that consists of clouds/IoT-edges (V), and (radio) access nodes ((R)AN) U. Meanwhile, E and W represent the links between V∪U, and their characteristics including the delay and the bandwidth capacity between distinct nodes, respectively. For the sake of simplicity, we consider overlay network, such that E is the end-to-end connections between V∪U. We consider $W=(W^{B},W^{L},W^{S})$, such that $W^{B}$, $W^{L}$ and $W^{S}$ are the bandwidth capacity, propagation delay and security level of the links in E. We assume that the managed IoT system supports a set of vertical industries, each of which has different requirements, such as Ultra-Reliable Low latency Communications (URLLC) and Enhanced Mobile Broadband (eMBB). Let Γ denote the set of verticals in the system, whereby each vertical γ∈Γ uses a set of similar service function chaining (SFCs). Each vertical γ∈Γ has a specified characteristic vector $\Sigma_{\gamma }$ that shows the end-to-end delay, bandwidth and security level requirements of that vertical, respectively. Let $\Sigma_{\gamma }^{L}$, $\Sigma_{\gamma }^{B}$ and $\Sigma_{\gamma }^{S}$ denote the end-to-end delay, network bandwidth and security level, respectively.

In network slicing, each vertical γ∈Γ should use a set of SFCs to handle the traffic of that vertical while ensuring network isolation and the targeted Key Performance Indicators (KPIs). Let $\Theta_{\gamma }$ denote the set of SFCs of the vertical γ. Moreover, each vertical γ has a set of UEs that use its offered services. Let $\Phi_{\gamma }$ denote the set of users and IoT devices of the vertical γ. Each user or IoT device $\phi \in \Phi_{\gamma }$ is expected to generate an amount of traffic $\lambda_{\phi }$. Each a cloud/edge u∈V is characterized by a limited storage and computation resources including CPU, RAM and DISK. Let $\Delta_{u}$ a vector that shows the resources of the cloud u∈V. Let R denote the resources targeted in this paper that could equal to {RAM,CPU,DISK}. We denote by $\Delta_{u}^{r}$, for r∈R, the amount of resource *r* available at the cloud *u*.

### 4.2. Security Orchestrator Optimizer

In the proposed model, we leverage the strength of SDN technology to interconnect the User Equipment’s (UE) $\Phi_{\gamma }$ (e.g., IoT devices) of the vertical γ to the network. The communication between a UE and its service happens through a specified SFC $\theta \in \Theta_{\gamma }$ that consists of a list of VNFs $\Upsilon_{\theta }$. As we have mentioned in the previous section, each VNF has a specified type, such as firewall, load balancer, … etc. We denote by Π the set of VNFs types supported in the system. We denote by $\pi_{\upsilon }$ the type of the VNF $\upsilon \in \Upsilon_{\theta }$, such that $\pi_{\upsilon }\in \Pi$. The VNFs $\Upsilon_{\theta }$ consists of three parts:

- The ingress point ($\upsilon_{I}\in \Upsilon_{\theta }$), which is the first VNF in $\Upsilon_{\theta }$. The user and IoT devices initially attache to (R)AN, and then from the (R)AN to the ingress point ($\upsilon_{I}$);
- The egress point ($\upsilon_{E}\in \Upsilon_{\theta }$), which is the last VNF in $\Upsilon_{\theta }$.
- The intermediate VNFs ($\upsilon \in \Upsilon_{\theta }\backslash \left\{\upsilon_{I},\upsilon_{E}\right\}$);

We denote by $\left(\upsilon_{i},\upsilon_{j}\right)\in \Upsilon_{\theta }$ a two consecutive VNFs in the SFC θ∈Θ. The latter is defined through a network slice blueprint that describes how the network functions (VNFs, VSFs and PNFs) interact among themselves for achieving a common objective and specific KPIs. The network slice blueprint also describes the needed resource for each network function. This template could be generated and maintained using different tools such as TOSCA (http://docs.oasis-open.org/tosca/tosca-nfv/v1.0/csd04/tosca-nfv-v1.0-csd04.pdf) and OSM model (VNFD and NSD) (https://osm.etsi.org/wikipub/index.php/OSM_Information_Model). In the blueprint, the KPIs of each SFC θ∈Θ are also defined in terms of end-to-end delay and bandwidth, as well as the security levels of the links used for interconnecting θ’s components. While the two former metrics target the Quality of Experience (QoE), the latter targets the security level of θ. Let $\sigma_{\theta }^{L}$, $\sigma_{\theta }^{B}$ and $\sigma_{\theta }^{S}$, denote the θ’s end-to-end delay, bandwidth and security level, respectively.

Two SFCs $\theta_{1},\theta_{2}\in \Theta_{\gamma }$ of the same vertical γ should have similar VNFs in the same order. Formally, $\Upsilon_{\theta }=\left\{\upsilon_{I},\cdots \upsilon_{E}\right\}$. For a SFC $\theta \in \Theta_{\gamma }$, let denote by $\left(\upsilon_{i},\upsilon_{j}\right)\in \Upsilon_{\theta }\times \Upsilon_{\theta }$ an order list, such that $\upsilon_{j}$ is the successor of $\upsilon_{i}$ in the SFC $\Theta_{\gamma }$.

In what follows, we will summarize the end-to-end delay, bandwidth and security ensured by different SFCs, respectively. In fact, the communication, between different clouds, VNFs, users and IoT devices, is characterized by different delay, bandwidth, jitters and security levels. For instance, the communication link within the same cloud has a higher security level while the one between users, IoT devices and inter clouds that do not use security mechanisms, such as IPSEC, SSL, or Datagram Transport Layer Security (DTLS), has the lowest security level. Each SFC is created from a predefined blueprint that specifies its characteristics and targeted KPIs that should be respected in terms of link security level, QoS, delay and bandwidth as mentioned before $\sigma_{\theta }^{L}$, $\sigma_{\theta }^{B}$ and $\sigma_{\theta }^{S}$, respectively.

In each SFC $\theta \in \Theta_{\gamma }$, the end-to-end delay is affected by the following parameters:

1. Service delay: This parameter considers the processing time needed by the destination VNF to process the data sent by the source VNF. The more resources at the destination VNF are, the lower service delay should be expected. The service delay of a VNFI v∈V can be computed using $F(v,\delta_{v})$. The amount of resources $\delta_{v}$ used by VNFI *v* has a positive impact on the service delay.
2. Propagation Delay: This delay refers to the time needed for traveling a single bit from the source VNF to the destination VNF. This delay varies according to distinct parameters including the distance between the clouds hosting these VNFs, as well as the quality of the link between these clouds. For instance, usually, the encrypted links would have lower delay and QoS. The encryption/decryption (e.g., VPN connection) of different packets harms the delay and the throughput, and hence low QoS will be perceived. In our model, if two VNFs belong to the same cloud/IoT-edge, then the propagation delay between them should be close to zero;

The outputs of the security orchestrator optimizer Algorithm are:

**Virtual network functions instances (VNFIs)***V*: The set of VNFIs that should be deployed and interconnected to serve different SFCs $\Phi_{\gamma }$ for γ∈Γ;
$\alpha_{\upsilon }^{r}$: denotes VNF resources r∈R used by the VNFI $\upsilon \in \Upsilon_{\theta }$;
$\delta_{\upsilon }$: The set of resources r∈R used by the VNF $\upsilon \in \Upsilon_{\theta }$;
$X_{\upsilon ,u}$: A decision variable that shows if the VNF υ uses the VNFI *u*;
$X_{\upsilon ,c}$: A decision variable the shows if the VNF υ is deployed at the cloud *c*;
$Y_{u,c}$: A decision variable that shows if the VNFI u∈V is deployed at the edge/cloud c∈V;
$T_{c1,c2}$: A variable that denotes the propagation delay between cloud/edge c1∈V and c2∈V;
$T_{u}$: A variable that denotes the service delay of the VNFI *u*;
$T_{\upsilon }$: A variable that denotes the service delay of the VNF υ;
$T_{\upsilon_{1},\upsilon_{2}}$: A variable that shows the propogation delay between $\upsilon_{1}$ and $\upsilon_{2}$.

Formally, there is relationship between $X_{\upsilon ,u}$, $X_{\upsilon ,c}$ and $Y_{u,c}$, respectively.

$$\begin{array}{c} \forall c\in V,\forall \gamma \in \Gamma ,\forall \theta \in \Phi_{\gamma },\forall \upsilon \in \Upsilon_{\theta }: \end{array}$$

(1) $$\begin{array}{cc} \; & X_{\upsilon ,c}\leq \sum_{u\in V}X_{\upsilon ,u}\times Y_{u,c} \end{array}$$

(2) $$\begin{array}{cc} \; & \forall u\in V:X_{\upsilon ,c}\geq X_{\upsilon ,u}\times Y_{u,c} \end{array}$$

We have also the following statements should hold:

(3) $$\begin{array}{cc} \forall r\in R,\forall u\in V: & \delta_{u}^{r}=\sum_{\gamma \in \Gamma }\sum_{\theta \in \Phi_{\gamma }}\sum_{\upsilon \in \Upsilon_{\theta }}\alpha_{\upsilon }^{r}\times X_{\upsilon ,u}\; \end{array}$$

(4) $$\begin{array}{cc} \forall r\in R,\forall c\in V: & \sum_{u\in V}\delta_{u}^{r}\times Y_{u,c}\leq \Delta_{c}^{r} \end{array}$$

The statement (3) ensures that the sum of resource *r* used by the VNFs υ equals to the resources of VNFI *u* where those VNFs are hosted. Meanwhile, the statement (4) ensures that the resources *r* of the VNFIs u∈V that are deployed in the cloud *c* do not exceed its capacity.

In what follow, we will define the constraints that ensure end-to-end delay of each SFC is respected. Thus, the end-to-end delay of each SFC $\theta \in \bar{\Theta }$ does not exceed its threshold $\xi_{\theta }^{L}$.

First of all, the outputs of the Algorithm is the processing delay of the VNFI $T_{u}$ and VNF $T_{\upsilon }$, respectively. While equality (5) presents $T_{u}$, the equality (6) shows $T_{\upsilon }$.

(5) $$\forall u\in V:T_{u}=\sum_{\gamma \in \Gamma }\sum_{\theta \in \Phi_{\gamma }}\sum_{\upsilon \in \Upsilon_{\theta }}F\left(u,\delta_{\upsilon }\right)$$

(6) $$\forall \gamma \in \Gamma ,\forall \theta \in \Phi_{\gamma },\forall \upsilon \in \Upsilon_{\theta }:T_{\upsilon }=X_{\upsilon ,u}\times T_{u}$$

Then, we need to compute the propagation delay from a VNF $\upsilon_{i}$ to its successor $\upsilon_{j}$ in the same SFC $\Upsilon_{\theta }$.

(7) $$\begin{array}{cc} \forall \gamma \in \Gamma ,\forall \theta \in \Phi_{\gamma }, & \left(\upsilon_{1},\upsilon_{2}\right)\in \Upsilon_{\theta }^{2}:\\ T_{\upsilon_{1},\upsilon_{2}}=\sum_{c1,c2\in V}X_{\upsilon_{1},c1}\times X_{\upsilon_{2},c2} & \frac{1}{W_{c1,c2}^{B}}\underset{\left(7.a\right)}{\underset{︸}{\sum_{\gamma \prime \in \Gamma ,\phi \in \Phi_{\gamma \prime },\theta \prime \in \Phi_{\gamma \prime },\left(\upsilon \prime_{1},\upsilon \prime_{2}\right)\in \Upsilon_{\theta \prime }^{2}}\lambda_{\phi }\times X_{\upsilon \prime_{1},c1}\times X_{\upsilon \prime_{2},c2}}} \end{array}$$

The part (7.a) shows the amount of traffic exchanged between the cloud c1 and c2.

Last but not least, we have to ensure that the end-to-end delay for each SFC θ is respected and do not exceed the required threshold $\xi_{\theta }^{L}$.

(8) $$\begin{array}{c} \forall \gamma \in \Gamma ,\forall \theta \in \Phi_{\gamma }:\underset{\left(8.a\right)}{\underset{︸}{\sum_{\upsilon \in \Upsilon_{\theta }}T_{\upsilon }}}+\underset{\left(8.b\right)}{\underset{︸}{\sum_{\left(\upsilon_{1},\upsilon_{2}\right)\in \Upsilon_{\theta }^{2}}T_{\upsilon_{1},\upsilon_{2}}}}\leq \sigma_{\theta }^{L} \end{array}$$

While the part (8.a) presents the computation delay expected by an SFC θ∈Θ, the second part (8.b) presents the propagation delay expected in the SFC θ.

Finally, we need to ensure that all the paths selected by the VNFs of a given SFC respect the constraints of security. Indeed, all the links that interconnect VNFs of the same SFC θ∈Θ should have a security level higher than the security level $\xi_{\theta }^{S}$.

(9) $$\begin{array}{c} \forall c1,c2\in V,\forall \theta \in \Theta ,\forall \left(\upsilon_{1},\upsilon_{2}\right)\in \Upsilon_{\theta }^{2}:\sigma_{\theta }^{S}\times X_{\upsilon_{1},c1}\times X_{\upsilon_{2},c2}\leq W_{c_{1},c_{2}}^{S} \end{array}$$

## 5. Semantic-Aware and Policy-Based Orchestration in IoT Systems

Security policies are intended to allow administrators to manage the system behaviour at a high level of abstraction by providing important features like flexibility and interoperability, tackling IoT challenges like massive deployments, heterogeneity and vendor locking since policy models are independent of the underlying infrastructure technologies. Specifically our framework provides two levels of abstraction for policy modeling [26]. The first one, defined in a High-level Security Policy Language (HSPL) allows to define security behaviours at a high level, mostly referring to subjects, actions and conditions as human readable names. In this way even a non-technical user can manage some security aspects of the system without specific knowledge of the current deployment. The second one, defined in a Medium-level Security Policy Language (MSPL), allows to specify security parameters in more detail than the previous one but still independent on the underlying technologies. Thus, MSPL allows to provide specific techniques, protocols, network information etc. Since the security behaviour can be modeled at different levels of abstraction, the framework provides a policy interpreter in order to refine HSPL policies into MSPL policies, and finally to translate MSPL policies in final end-points configurations. While the refinement process is focused on transforming the high level concepts into the real values of the infrastructure deployment, e.g., to transform a service name to a specific IP, protocol and port, the translation process is in charge of generating specific configuration for the specific endpoint where the policies will be enforced, e.g., to generate SDN rules or iptables configurations [27]. Although one security policy can be enforced as independent proactive or reactive countermeasure, the aforementioned security policy languages have been evolved in order to provide orchestration policies.

In this paper we introduce the Security Orchestration Policies, intended to not only embrace a set of security policies, but also to model other important aspects such as enforcement priority and dependencies among them, where dependencies indicate that a security policy can be conditioned to the enforcement of another security policy or even it can depend on the fulfilment of an specific event. In this way, a security administrator is able to model more complex behaviours like, for instance, security policy enforcement orchestration plan by specifying a combination of networking and service configuration security policies.

### 5.1. Policy-Based Security Orchestration in IoT, Motivation Example Scenario

To motivate the need of security orchestration in IoT systems, let us assume we want to orchestrate the bootstrapping behaviour of IoT devices. During bootstrapping, the new IoT device will attempt to connect in the IoT domain for the first time. To this aim, it will first try to connect with the IoT Controller entity or Authentication Agent (deployed as VSF) in charge of configuring the authentication parameters including IoT authentication method e.g., PANA (Protocol for Carrying Authentication for Network Access) with pre-shared keys (PSK) popular in IoT scenarios). Once authenticated, the IoT device should be given rights, at application level, to access or push data in specific resources (e.g., in an IoT Context broker). Furthermore, at network level, the IoT management system should allow, through SDN, the corresponding traffic from the IoT device to the Context broker and the IoT Controller and viceversa.

**Figure 3:**
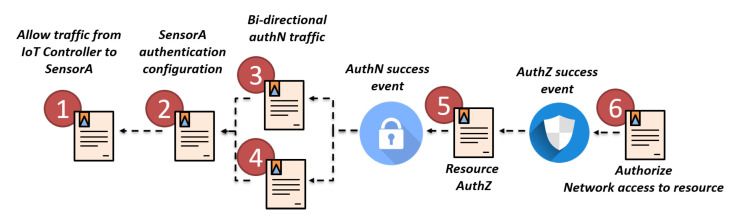
Policy dependencies example during Security orchestration.

That example can be modelled through an orchestration security policy composed of several authentication, authorization and filtering policies. In this sense, Figure 3 shows the aforementioned example of authentication and authorisation policy for orchestration in an IoT environment. In this case, the orchestration policy allows first the communication between the IoT controller and the IoT device specified as *SensorA* (Figure 3 step 1). When this first security policy has been properly enforced, an enforcement event is raised so the policies which depend on it can be enforced. In this case, the IoT Controller is able to configure the *SensorA* by indicating specific authentication parameters (Figure 3 step 2) since the traffic against the *SensorA* is now allowed. Once the IoT device has been properly configured, the specific authentication protocol must be allowed in the network (Figure 3 steps 3 and 4). Regarding the authorization policies, the authorization will be performed only when the device has been properly authenticated, then in this example the resource authorisation policy depends on the authentication success event. When the authentication success is received the authorisation policy will be enforced by configuring authorization parameters in a Policy Decision Point (Figure 3 step 5). Finally, when the system receives the authorisation success event, the specific network traffic for the specific authorised resource will be allowed (Figure 3 step 6).

Listing 1 shows an excerpt of the orchestration policy that models the behaviour of the example shown in Figure 3. As it can be seen in the policy, it is comprised of six security policies needed to accomplish the security orchestration behaviour. Each policy has his priority value to order them, and the dependencies associated in order to condition the deployment of the policies to the fulfilment of particular events.

**Listing 1:**
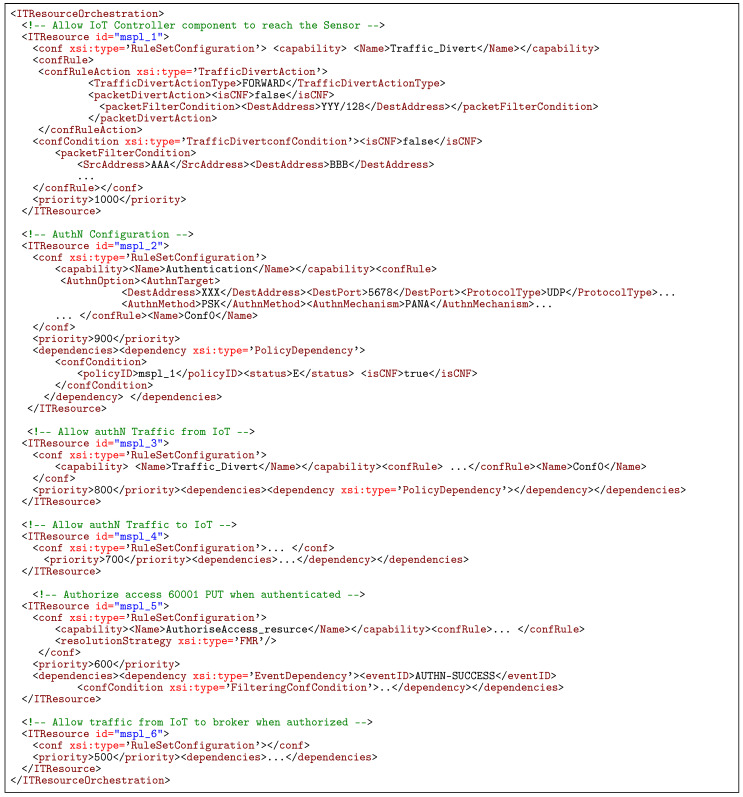
Example of Security Orchestration policy to manage the bootstrapping in IoT systems.

### 5.2. Policy-Based and Contextual Automatic Reasoning Process

The Policy Interpreter is endowed with a reasoner engine that allows to infer automatically new valuable information that was not initially defined in the system model. The reasoning is done by performing a formal validation and verification of the system model and policies, using logic programming language, where the logic is expressed in terms of relations which, in turn, are represented as rules and facts.

The Description Logics (DL) formalism on which these logic programming languages are based, is kept within the decidability bounds, so that inference processes are performed in a finite time. The system model is kept up to date in a Knowledge Base (KB) that holds contextual and system information from the underlying managed IoT system, and there is a process to transform dynamically those system model’s concepts in facts understandable by the rule-based reasoner engine.

The logic rules are formed by antecedent representing the Conditions of the tuple and the consequent, executed when the antecedent is fulfilled. The rules make reference to the MSPL security policy statements and the associated concepts. Moreover, the rules can make reference to elements of the system model and other metrics used as conditions. Thus, only when the conditions are met, the consequent is triggered. This allows adding additional facts when the certain contextual and policy conditions are met. These facts are hold in the Knowledge Base managed by the Policy Interpreter.

A set of semantic rules are defined to detect in the antecedents conflicting behaviours referring to facts in security policies and system model (KB). If the antecedent of the rule is met the consequent of the rule can force a conflict by including contradictory facts in the Knowledge Base that are detected, as conflicts, by the reasoner. The rule reasoning process allows detecting automatically contradictions in both, MSPL and Orchestration policies, as well as detecting semantic conflicts in the managed system.

### 5.3. Semantic Conflict Detection in Policy-Based Security Orchestration

During the orchestration policy enforcement it becomes necessary to detect any kind of incompatibility, contradiction or dependency prior enforcing the policies as system configurations for Virtual Security Functions (VSF) and SDN rules. In that sense, semantic conflicts or dependencies can occur in the same orchestration policy (intra-orchestration) or between orchestration policies (inter-orchestration). In our proposal this kind of conflict detection is performed by the policy conflict detection process based on a rule engine.

The following subsections are intended to provide several kind of semantic conflicts and dependencies associated to the security orchestration and management process, as well as corresponding semantic rules aimed to detect those conflicts.

#### 5.3.1. Redundancy Conflict in Policies

The conflict detector verifies whether there is another security policy with the same identifier or with the same behaviour. Rule 1 shows an example of conflict detection rule, when a same behaviour is shared for more than one security policy.

Rule 1: Same Behaviour Conflict example.

 


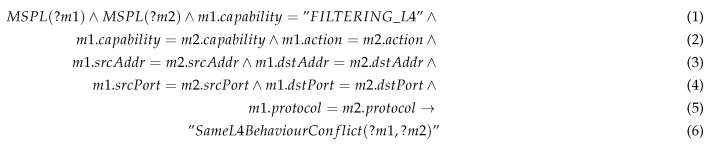


Specifically, this example shows a rule aimed to detect the same behaviour on level 4 filtering capability. This is, there are two any different policies, MSPL *m1* and a MSPL *m2* with the filtering capability for the same networking configuration up to level 4. This kind of conflict might be raised when two security administrators, belonging to the same organisation receives simultaneously an alert regarding unauthorised traffic against a specific service (e.g., CoAP server), then both decide to apply CoAP filtering policy with the same content. When one of the security policies has been registered, the second attempt will be detected as a same behaviour conflict. The consequence in the rule will generate a new fact in the KB, that generate an inconsistency in the reasoner. Besides, this process will empower the security administrators with means to verify those conflicts and notice whether the security policy has been already enforced.

#### 5.3.2. Priorities Conflict

This kind of conflict occurs during the Orchestration, when a security policy belonging to an orchestration policy, with lower priority is going to be enforced before another one with a highest priority. It also verifies if there a security policy depends on another with lower priority.

Rule 2 shows an example where both MSPL *m1* and *m2* have a established priority but *m1* has a higher one but it depends on the previous enforcement of *m2*. This kind of conflict might happen when a security administrator defines an Orchestration Policy aimed to turn off an IoT device and then filter the IoT traffic for the specific IoT device. The filtering policy depends on the IoT control policy since if the filtering policy is enforced first, the IoT Controller will not be able to communicate with the IoT device in order to turn it off. If the filtering policy is configured with higher priority than the IoT control one (e.g., due to a human error), the security administrator is notified with the priority conflict.

Rule 2: Priority dependency conflict

 


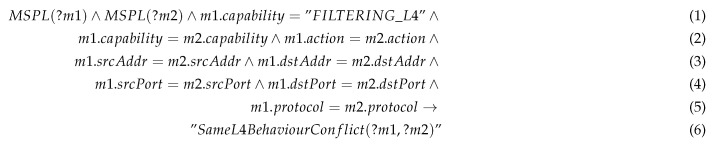


#### 5.3.3. Duties Conflict Across Policies

Duties conflict appears when the enforcement of the security policy can harm another security policy which is already deployed or which is going to be deployed. In order to detect this kind of conflicts, it is required to design a relationship between those capabilities which are not compatible.

Rule 3 shows an example of duties conflict where the MSPL *m1* is intended to configure the traffic monitoring but *m2* aims to configure channel protection for the same source and destination. A security administrator enabled Deep Packet Inspection between a pair of endpoints, and the same administrator or another one decides to apply an IoT channel protection policy using DTLs protocol for the monitored communication channel. In this point the system administrator will be notified with a conflict of duties since if the traffic is encrypted the DPI process will be affected.

Rule 3: Duties dependency conflict

 


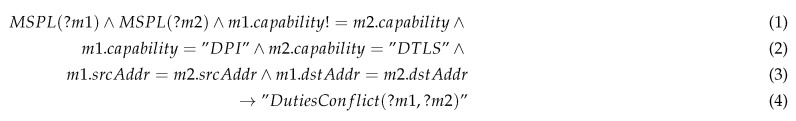


#### 5.3.4. Event and Policy Dependencies

A security policy belonging to an Orchestration Policy can require that a previous one or a set of previous ones have been already deployed before it can be enforced. On the other hand, the enforcement of some kind of security policies only make sense after specific processes have been finished (e.g., Authorisation after authentication).

Rule 4 shows both, an example where MSPL *m1* has a dependency which is the type *EVENT* as well as the *POLICY* event type. This conflict might occur when a security administrator models an Orchestration Policy which, in turn, includes forwarding, authentication and authorisation policies. The forwarding policies will connect the authentication traffic with the authentication entity. The authentication policies will configure the authentication of the IoT devices but these policies depend on that the forwarding policies are enforced (policy dependency). On the other hand, the authorisation policies will allow access to specific resources once the device authentication has been performed successful, so in this case authorisation policies will depend on a success authentication event (event dependency).

Rule 4: Event dependency conflict

 


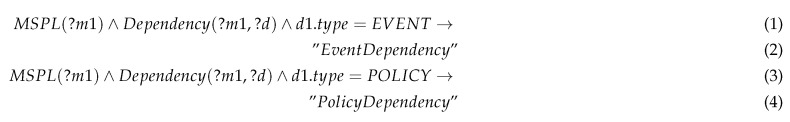


#### 5.3.5. Managers Conflict

When the system behaviour depends on several administrators, a managers conflict can appears. In fact, it can also happen for the same administrator at different time instants. In this case, one manager desires to enforce some configuration which contradicts another previously enforced or which is in the same orchestration policy.

Rule 5 shows an example of a managers conflict rule in order to detect conflicts for the filtering capability up to l4. The MSPL *m1* allows a specific kind of traffic whereas *m2* aims to deny it. A security administrator decides that the traffic coming from a specific IoT device must be dropped since it seems suspicious but at the same time, other security administrator decides to forward the same kind of traffic to an IoT honeynet. In this case the security administrator will be notified with a managers conflict.

Rule 5: Managers conflict

 


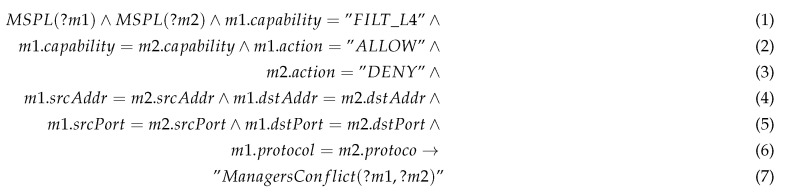


#### 5.3.6. Override Conflict in Orchestration

A security policy could override the behaviour of another one for instance by being more permissive than the previous one.

Rule 6 shows an example where the MSPL *m1* aims to perform the same action than *m2* but it is more permissive since it allows all the traffic for the source address. For instance, *m2* could allow Constrained Application Protocol (CoAP) traffic from an IoT device to a specific target and later *m1* aims to allow all the traffic coming from the IoT device. As an example of this conflict, a security administrator might decide to enforce a filtering policy in order to deny specific kind of traffic (e.g., CoAP traffic coming from an specific IoT device and directed to an specific CoAP server). Then the same administrator, or another one, tries to apply a security policy that includes the previous one (e.g., deny all CoAP traffic). In this case the security administrator will receive a notification with override conflict.

Rule 6: Override Conflicts conflict

 


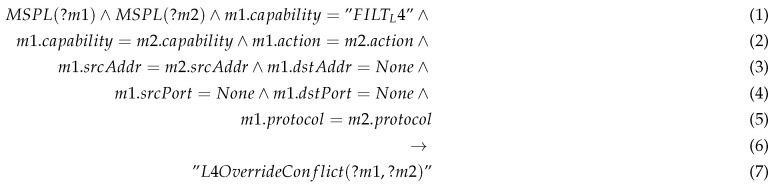


### 5.4. Context-Based Conflict Detection and Dependencies

While the semantic conflicts are focused on analyzing issues between security policies, the context-based conflict detection approach determines conflicts or dependencies by taking into account the whole infrastructure.

The following subsections explain some examples of context-based conflict detection and dependencies through semantic rules, that infer in the consequences some contradictory facts, that, when added to KB, allows the reasoner to detect inconsistency.

#### 5.4.1. Capability Missing Conflict

Capability missing conflict occurs when a specific capability enforcement is requested, but the element, who should enforce it, is not able to do it, e.g., It does not implement the specified capability.

As an example, let us consider a security administrator trying to enforce a channel protection security policy by introducing the specific security protocol he wants (e.g., DTLS) but the target IoT device does not implement that feature. It can be also applicable for deploying security enabler in a VNF that do not support the capability. Rule 7 shows an example of this kind of conflict.

Rule 7: Capability missing conflict example

 


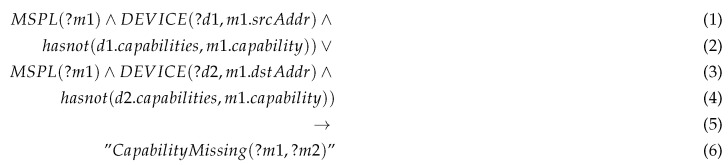


#### 5.4.2. Insufficient Resources Conflicts

This kind of conflict appears when the system tries to enforce a security policy which requires some available resources which are currently unavailable or insufficient.

Rule 8 shows the rule needed to detect insufficient resources, where security administrator tries to enforce different channel protection security policies in the same IoT device for different destinations and the IoT device has not enough resources in order to maintain the requested amount of protected channels simultaneously.

Rule 8: IoT Insufficient resources conflict detection example
MSPL

 


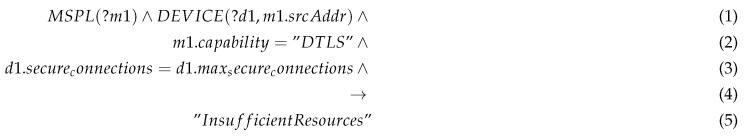


Similarly, although they are not included here for the sake of space, additional semantic rules are defined to check whether the security level calculated for the MSPL security parameters is higher than the one that can be implemented by the selected VNF.

### 5.5. Conflict Detection and Dependencies Process

**Figure 4:**
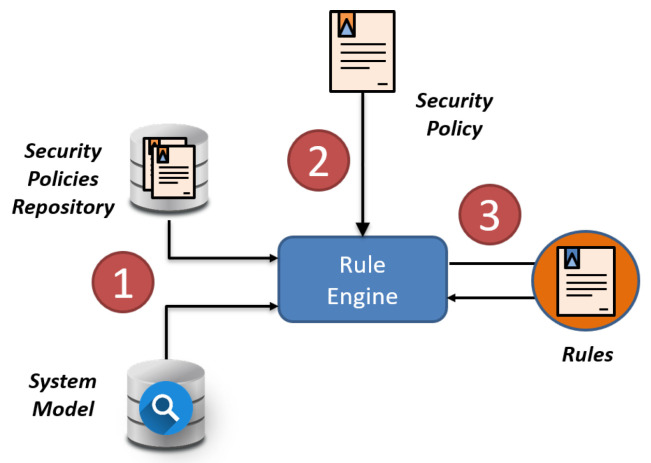
Rule engine representation.

The conflict detection and dependencies processes are carried out by the policy conflict detector, which implements a rule engine able to process different kind of security policies against a set of rules defined. Figure 4 shows a simplification of the rule engine. At start-up time, the rule engine compiles the defined rules and creates new facts for current security policies already deployed in the system as well as for current information of the deployment like VNFs or events (Figure 4 step 1). It is important to highlight that system model and policy updates are also reflected in the Knowledge Base of the rule engine dynamically so the rule engine remains always updated. Thus, when the rule engine receives a new security policy (Figure 4 step 2), it performs the rule matching against the latest known facts (Figure 4 step 3), which verifies if the new policies present any kind of conflicts or dependencies between them or between them and the system according to the current facts. The process then generates a list of tuples (O1,I,O2) where On represent the kind of object involved (e.g., Event or policy) and I is the kind of issue (e.g., Event dependency).

**Figure 5:**
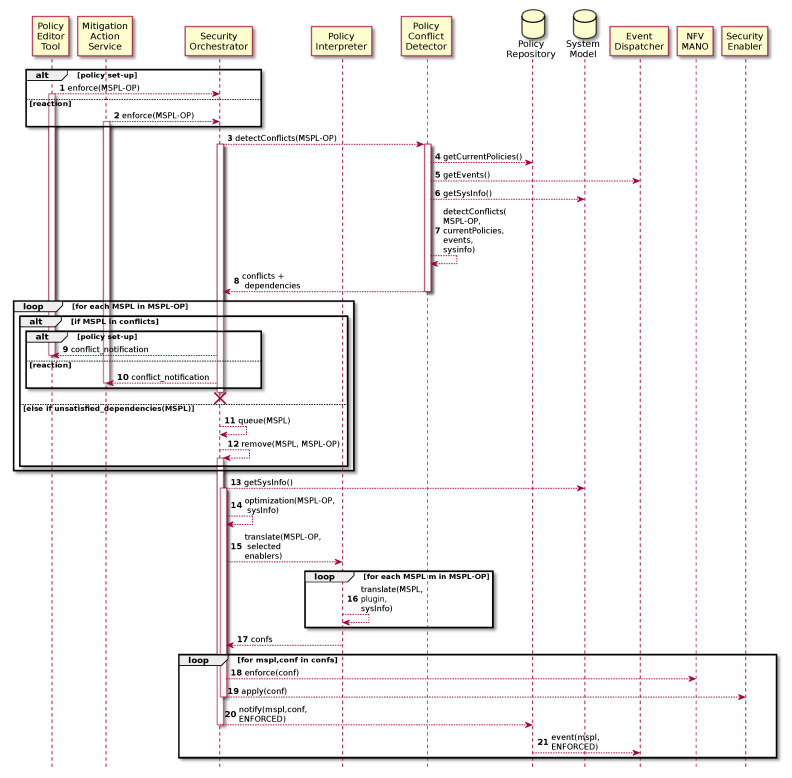
Policy Orchestration and Enforcement process.

The result of the conflict detection is used during the policy enforcement process in order to deploy the policies according to the specification and the current status of the system. The security orchestrator must manage properly the conflicts and dependencies in order to avoid including misbehaviour in the system. Figure 5 shows the steps involved in the policy enforcement taking into account conflicts and dependencies management. Since the orchestration policy enforcement can be either proactive or reactive, the enforcement process can be triggered by the administrator through the Policy Editor Tool or by the system through the Mitigation Action Service respectively (Figure 5 steps 1 and 2).

Once the Security Orchestrator receives the MSPL Orchestration Policy (MSPL-OP), it requests the conflict and dependencies detection to the Policy Conflict Detector (Figure 5 step 3). For the sake of simplicity, in this workflow Policy Conflict Detector retrieves current security policies already deployed in the system as well as available events and information for the subjects involved in the received MSPL-OP in order to generate the base of knowledge of the rule engine (Figure 5 steps 4–6). However, that information can by gathered dynamically by using an event subscription approach in order to keep the Knowledge Base properly updated. With this information, it performs the conflict and dependency detection and it returns the result to the Security Orchestrator (Figure 5 steps 7 and 8). The Security Orchestrator then verifies the results. The conflicts are notified to the administrator, or to the system depending on who started the enforcement process (Figure 5 steps 9 and 10) since security policies which present conflicts will not be enforced.

Once conflicting security policies have been notified, dependencies are analyzed and managed. Each dependent security policy must be queued until it is satisfied (Figure 5 steps 11 and 12). Rest of the policies as well as system model information are then provided to the optimization process described in Section 4 which decides the best security enabler in order to enforce the security policies (Figure 5 steps 13 and 14) as well as the best compute node to allocate the Virtual Security function (VSF). It is important to highlight that, as part of the optimization algorithm, the Security Orchestrator can request new context-aware policy conflict analysis, now considering security enabler candidates for the policy enforcement. Once security enablers have been selected, the Security Orchestrator requests the policy translation to the Policy Interpreter in order to obtain the proper configurations for each security enabler (e.g., a VSF) involved in the policies enforcement (Figure 5 step 15). Policy Interpreter verifies that the MSPL-OP is well formed and then it translates each MSPL in the MSPL-OP by using the specific security enabler plugin which was previously selected by the Security Orchestrator, providing then a matching among MSPLs, and final security enabler configurations to the Security Orchestrator (Figure 5 steps 16 and 17). Security Orchestrator performs NFV-MANO operations like new enablers deployment if required (Figure 5 step 18) and each configuration is enforced by using specific drivers which implements the way of communicate the final configurations to the specific security enabler (Figure 5 step 19). Finally, Policy Repository registers the new status of the security policies which will be also reflected as a new event which will be taken into account by the Event Dispatcher (Figure 5 steps 15 and 16).

**Figure 6:**
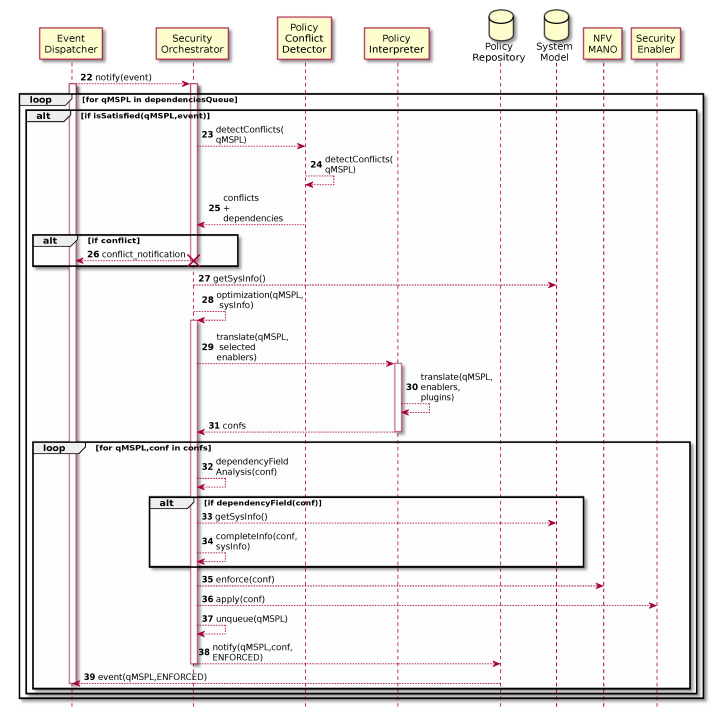
Dependencies management example.

The Event Dispatcher is in charge of receiving different events of the system and provide them across the different components subscribed to the specific event which in fact could trigger dependencies management processes. Figure 6 shows the workflow for dependencies management. When the Event Dispatcher notifies an event to the Security Orchestrator, it verifies if the event satisfies the dependency of any of the queued security policies. When a security policy is satisfied, Security Orchestrator must ensure that the satisfied security policy is still valid for the system, so it starts a new policy conflict detection process by using the Policy Conflict Detector (Figure 6 steps 23–25). If there is any kind of conflict, it is notified and the policy is not enforced (Figure 6 steps 26). Otherwise, Security Orchestrator retrieves system model information, decides the best security enabler by applying the optimization process and performs the pertinent translations through the Policy Interpreter (Figure 6 steps 27–31). Once it receives the final configurations, it completes missing information in the security policy if required (e.g., a VM IP address that was not already launched at the timing the dependency was detected but now that information is available) (Figure 6 steps 32–34). Finally, the Security Orchestrator enforces the configurations in the same way of the previous explanation, but in this case it also unqueues the security policy, since the dependency has been solved.

It is noteworthy that the received events are not only related to other security policies but system events like authentication or authorization results. For instance, an authorisation security policy could depend on the result of the authentication process. In this case the Event Dispatcher would send the authentication success event to the Security Orchestrator who will verify that the authorisation which depended on the authentication success event is now satisfied.

## 6. Validation and Performance Evaluation

Regarding the implementation, Policy Editor Tool, Policy Interpreter (Refiner and Translator), System Model and Security Enablers Provider components were implemented in different Python3 frameworks such as Django, Django-rest and falcon. Mitigation Action Service were provided in Java by Montimage company. Previous components, except Mitigation Action Service, were dockerised and deployed in an Intel Core i7-7700 at 3.60 GHz with 8 GB of RAM. The Policy Conflict Detector engine has been implemented in Python3, using the rule engine Pyke version 3.1. Pyke is a knowledge-based inference engine (expert system) that allows to apply rules over facts kept in the KB, thereby establishing additional facts, this allow as to define the rules of Section 5.3 to detect conflicts in the security and orchestration policies.

### 6.1. Policy Conflict Detection in Orchestration Policies

In order to validate the orchestration conflict and dependencies detection we have measured the time it takes for the conflict detector to detect conflicts and dependencies. Along the different executions we provide as input different types of orchestration security policies (forwarding, dtls, authorization and AAA) which are composed by different amount of policies (2, 2, 4 and 6 respectively). We fixed the system model facts in order to represent 500 facts regarding the VNFs information and we vary the current enforced security policies in the system from 0 to 1000 as well as the quantity of available rules in the rule engine. Each combination of variables (policy input, system model policies and rules) was executed 60 times and each bar in the figures shows the mean as well as the 95% confidence interval in seconds. In this way, for each execution, each security policy enclosed in the orchestration policy is verified against each rule. Depending the nature of the rule, it verifies security policy values against two different types of facts, these are, the VNFs system model information and the policy enforced system model information. The provided results do not include the time taken by the system for loading the facts to the system model, which is done just once at start-up, and in the most expensive use case (1000 policies, 500 VNF facts) it takes around 4 min.

**Figure 7:**
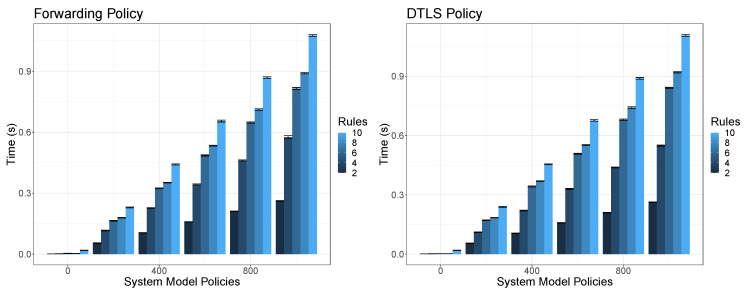
Conflict detection performance of network forwarding and DTLS policies according to the rules.

Figure 7 shows the time taken by the conflict detector to detect conflicts and dependencies in a bi-directional forwarding policy and a bi-directional DTLS policy, varying the rules from 2 to 10 as well as the amount of already enforced security policies from 0 to 1000. In this case the results are quite similar since both orchestration policies are composed by two policies which are also similar in terms of complexity. In fact, DTLS is a little bit complex and that is the reason why the times are slightly higher in that case. As it is expected, the time increases according on the number rules and the amount of already enforced policies since the input policies must be compared with each of them (or each VNF fact) for each rule. It is important to highlight that is not only important the number of rules but also the complexity of each rule. This is, the rule that verifies if the policy identifier is unique in the system is simpler than the one that verifies if an specified VNF will be able to enforce the specified security level for a DTLS policy. This is the reason why although there is an increase of time for each new pair of rules, there is not a clear progression between them. In this case the time the system takes to perform conflict and dependencies detection for two security policies by considering 1000 already enforced policies, 10 different rules and 500 VNF system model facts is near to 1 s.

**Figure 8:**
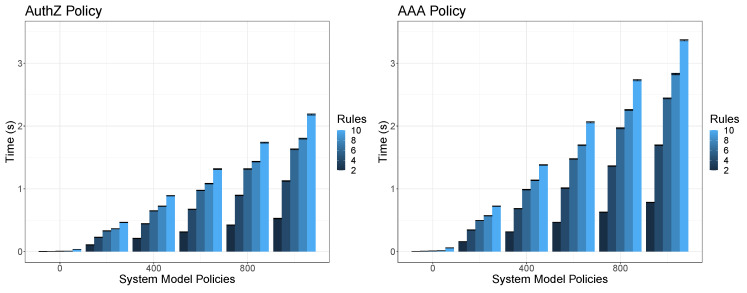
Conflict detection performance of Authorisation and AAA policies according to the rules.

Unlike the previous case, Figure 8 shows the results for an authorisation orchestration policy composed by 4 policies (two for authorising two different resources in a specific endpoint and 2 bi-directional forwarding policies which depends on the success authorisation event in order to allow the network traffic) and an AAA orchestration policy composed by 6 policies (allow the traffic from the IoT controller to an IoT device, configure authN in IoT device, allow bi-directional authN traffic, authorise access to two different resources when the previous authN process has been completed properly). While the number of rules and policies in the system model have a similar impact than in the previous cases, now the increase of the input security policies also increases the results from around 1 s per each pair of new security policies. This is, for the case with 6 security policies that are verified against 1000 security policy facts, 10 rules and 500 VNF facts the time increases up to 3.5 s. Of course, it is important to take into account that we consider 1000 security policy facts and 500 VNF facts as a complex use case so in a medium-size organisation the results could be improved. Despite it is out of the scope of this paper, for the sake of completeness, it is worth mentioning that policy refinement, translation and enforcement processes were addressed on previous works such as [4,5] providing average results in terms of centiseconds and deciseconds depending on the kind of enforced policy and the selected security enabler.

### 6.2. Security Orchestrator Optimizer Implementation and Evaluation

In this section, we evaluate the performances of the security orchestration optimizer mechanism. We have implemented and evaluated the security orchestrator optimizer using Python language and Gurobi Optimizer software. All the execution time measurements are based on a Dual Intel Xeon E5-2680 v3 @ 2.5 GHz, with 256 GB of RAM, and running Ubuntu 16.04. In all the simulations, we have considered 15 security levels, where we have uniformly distributed them among the connection links that interconnect clouds, edges and IoT domains. We have evaluated the suggested solution in different scenarios by running 35 repetitions. The plotted results present the mean and 95% confidence interval. In the evaluation, we have considered the following metrics:

- End-to-end delay: is defined as the average end-to-end delay, which includes the propagation and processing delays, perceived by different SFCs in different epochs.
- The cost: This metric has been presented as the total number of deployed VNFIs for serving different SFCs.

The security orchestrator optimizer has been evaluated by varying the number of edges and the number of deployed SFCs. We have evaluated our solution by fixing the number of SFCs by 10 while varying the number of edges/clouds.

**Figure 9:**
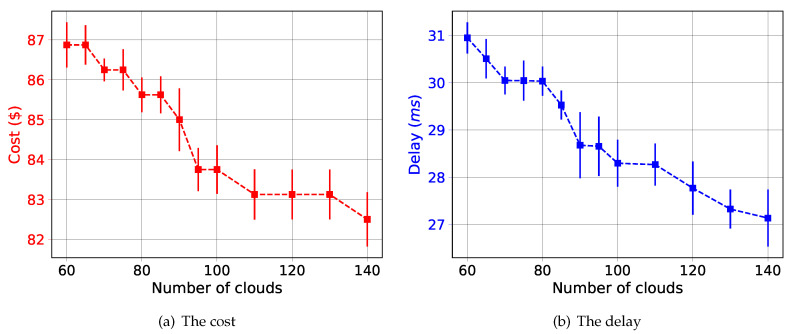
Cost and Delay of the security orchestrator.

Figure 9a depicts the impact of the number of clouds/edges on the cost. The first observation that we can draw from this figure is that the number of edges/clouds has a positive impact on the cost. Increasing the number of edges/clouds gives more flexibility to the solution for finding a better position for deploying the VNFIs that serve more VNFs, and hence helping more SFCs. This leads to reducing the number of VNFIs should be implemented, and therefore reduce the cost. Meanwhile, Figure 9b shows the performances of security orchestrator in terms of end-to-end delay while varying the number of edges/clouds. The first observation that we can draw from this figure is the increase in the number of edges/clouds has a positive impact on the end-to-end delay. The delay proportionally decreases with the number of edges/clouds in the network. Increasing the number of edges/clouds leads to increasing the likelihood of finding better locations for deploying different VNFIs that could offer better end-to-end delay for different SFCs.

## 7. Conclusions

This paper has presented a semantic-aware and policy-based security orchestration framework for SDN/NFV-aware IoT systems. The system automatizes and optimizes the security policy orchestration according to the actual context, using Semantic-aware technologies for checking any inconsistencies that might occur between the evolving IoT system model and instantiated security policies.

The conducted experiments show the feasibility and performance of the solution to handle several kind of conflicts in orchestration, and detect them in diverse kind of security policies such as filtering/forwarding, channel protection (DTLS), Authorization or AAA policies. The system has been stressed considering up to 1000 concurrent enforced policies, achieving affordable reasoning times for conflict detection and consistency checking, following a linear incremental trend as the number of policies increases.

Likewise, our policy-based security orchestration is endowed with a novel orchestrator optimizer aimed to automatically and optimally allocate the VSFs, considering several aspects during the optimization, such as the current network topology status, QoS in network, actual computing resources and security aspects. The results show that the number of edges/clouds has a positive impact on the end-to-end delay and cost. Increasing the number of edges/clouds increases the likelihood for funding better positions for deploying VNF instances that serve as many as possible SFCs with better KPIs. It hence leads to reduce the cost and the end-to-end delay.

As future work we envisage to evolve our orchestration optimizer to consider additional contextual requirements during the SFC chaining. Besides, we plan to improve our orchestration mechanism to embrace complex multi-tenant and multi-domain beyond 5G networks comprised of volatile and mobile virtual edge nodes.
